# A deep learning software tool for automated sleep staging in rats via single channel EEG

**DOI:** 10.1038/s44277-025-00035-y

**Published:** 2025-07-10

**Authors:** Andrew Smith, Snezana Milosavljevic, Courtney J. Wright, Charlie A. Grant, Ana Pocivavsek, Homayoun Valafar

**Affiliations:** 1 Computer Science and Engineering, University of South Carolina, Columbia, SC USA; 2 Department of Pharmacology, Physiology, and Neuroscience, University of South Carolina School of Medicine, Columbia, SC USA

**Keywords:** Sleep disorders, Computer science, Scientific data

## Abstract

Poor quality and poor duration of sleep have been associated with cognitive decline, diseases, and disorders. Therefore, sleep studies are imperative to recapitulate phenotypes associated with poor sleep quality and uncover mechanisms contributing to psychopathology. Classification of sleep stages, vigilance state bout durations, and number of transitions amongst vigilance states serves as a proxy for evaluating sleep quality in preclinical studies. Currently, the gold standard for sleep staging is expert human inspection of polysomnography (PSG) obtained from preclinical rodent models and this approach is immensely time consuming. To accelerate the analysis, we developed a deep-learning-based software tool for automated sleep stage classification in rats. This study aimed to develop an automated method for classifying three sleep stages in rats (REM/paradoxical sleep, NREM/slow-wave sleep, and wakefulness) using a deep learning approach based on single-channel EEG data. Single-channel EEG data were acquired from 16 rats, each undergoing two 24 h recording sessions. The data were labeled by human experts in 10 s epochs corresponding to three stages: REM/paradoxical sleep, NREM/slow-wave sleep, and wakefulness. A deep neural network (DNN) model was designed and trained to classify these stages using the raw temporal data from the EEG. The DNN achieved strong performance in predicting the three sleep stages, with an average F1 score of 87.6% over a cross-validated test set. The algorithm was able to predict key parameters of sleep architecture, including total bout duration, average bout duration, and number of bouts, with significant accuracy. Our deep learning model effectively automates the classification of sleep stages using single-channel EEG data in rats, reducing the need for labor-intensive manual annotation. This tool enables high-throughput sleep studies and may accelerate research into sleep-related pathologies. Furthermore, we provide over 700 h of expert-scored sleep data, available for public use in future research studies.

## Lay summary

Our study presents a new deep learning model for automatically analyzing sleep patterns in rats using EEG data. This model was trained on one dataset and tested on two others, showing it can adapt to different data, which highlights its generalizability. This advancement could streamline sleep research by reducing manual scoring, making it faster and more consistent, thus aiding in studies of sleep disorders and drug effects on sleep in rodents.

## Introduction

Sleep is essential for optimal health [1], yet over one third of the global population reports problems with sleep [2]. Preclinical sleep studies are imperative to recapitulate phenotypes associated with poor sleep and uncover mechanisms contributing to psychopathology [3–6].

Studies performed in rodents allow investigators to understand underlying mechanisms that drive sleep homeostasis [7]. Vigilance states that compose sleep are evolutionarily conserved amongst species; therefore, rodent models can recapitulate the unique polysomnographic characteristics of sleep states [8]. The accurate determination of vigilance stages into wake, rapid eye movement (REM) or paradoxical sleep, and non-REM (NREM) or slow-wave sleep is a critical step in analyzing acquired sleep-wake recordings. As REM and NREM sleep serve functions uniquely different from a wake state, correctly classifying vigilance states is imperative.

The gold standard for sleep staging in rodents is expert human inspection of waveforms generated from electroencephalography (EEG) and electromyography (EMG) signals. Unlike human polysomnography (PSG), which includes a broader array of signals such as EEG, EMG, electrooculography (EOG), and others, rodent sleep studies generally rely on fewer channels due to practical constraints. Manual annotation of PSG by human experts is time-consuming and requires extensive training limiting efficiency and failing to eliminate interscorer and intra-scorer variability [9]. Developing reliable automated rodent sleep staging methods is crucial for efficient preclinical sleep studies.

Tethered rodents are combating confounds including limited mobility within recording cages and potential impacts on natural sleep states [10–12]. Therefore, it is advantageous to collect PSG via surgically implanted transmitters. Due to the size and nature of available transmitters for collecting PSG, the number of signals acquired simultaneously during sleep is limited. Therefore, using a transmitter instead of tethering rodents via wires decreases the number of signals available and increases complexity of sleep staging [13].

Several published algorithms for rodent sleep staging require a combination of signals, such as EEG and EMG, which can be collected via telemetry transmitters [14]. In contrast, human sleep staging algorithms often incorporate additional signals, such as EOG [15], highlighting the need for rodent-specific algorithms that accommodate fewer channels. Therefore, developing an automated algorithm based on only a single-channel EEG signal is of interest because of its versatility across hardware and experimental settings. Further, automatic sleep staging based on a single-channel EEG signal collected via a transmitter would allow researchers the opportunity to investigate other signal types with remaining available channels, such as electrocardiogram (EKG).

Deep learning has revolutionized various fields, including time series analysis [16–20]. Advances in deep learning have garnered interest in developing automated algorithms for sleep staging. Several recent advancements in automated sleep staging are based on sleep studies in humans [15, 21–23] wherein sleep staging standards differ significantly from those in rodent sleep studies [7]. Nearly all methods for automatic sleep staging rely on multi-channel EEG recordings and derived local field potential (LFP) signaling oscillations within the brain [9, 24–28]. Of recently proposed deep learning algorithms for sleep staging, we have only come across two that have attempted automatic sleep staging in rodents via single-channel EEG [29, 30].

Human experts, after extensive training, rely on recognizing patterns in signals (like amplitude and frequency of EEG waves) to classify sleep epochs. Our model replicates this process using a convolutional neural network (CNN) to encode visual patterns in EEG signals into a simpler classification-friendly representation. This network processes each 10 s epoch independently, transforming raw signals into feature maps. The next step involves a Long Short-Term Memory (LSTM) network to capture the temporal dynamics of sleep, like how human experts analyze data by considering patterns over time. This combination of spatial and temporal encoding allows our model to not only classify sleep stages but also to understand the temporal context in which these patterns occur, significantly reducing the need for manual annotations and enhancing the efficiency of sleep studies.

In 2019, Miladinovic et al. introduced a CNN with a Hidden Markov Model (HMM) for automated sleep staging in rodents using their SPINDLE dataset. The authors report F1 scores ranging from 76 to 98 across four cohorts of data; however, these metrics were computed excluding artifacts. From the author’s confusion matrices provided, we estimate an overall F1 score between 91 and 94, including or excluding artifacts, respectively. These performances are based on a model taking 2 EEG and 1 EMG as input. The authors provide a comparison where they use 1 EEG and 1 EMG; however, they provide no comparisons without EMG. Since we are interested in sleep staging via only single-channel EEG and using end-to-end deep learning from raw data, whereas Miladinovic preprocesses data, we do not compare our work to theirs.

In 2021, Tezuka et al. developed a CNN-LSTM neural network for sleep staging in mice from only a single-channel EEG; however, the authors use a private dataset, constrained to the light phase of the light-dark cycle, and include Zeitgeber Time (ZT) and the Discrete Fourier Transformation (DFT) as input to their model [29]. The use of a private dataset limits researchers’ ability to confirm results or compare novel methods. Constraining data to the light phase of the light-dark cycle removes a dimension of variability that likely reduces the complexity of sleep staging [13]. Including ZT and DFT as input may introduce bias and limit generalizability.

In 2022, Liu et al. developed a CNN in serial with an attention mechanism for sleep staging in rodents from only a single-channel EEG [30]. The authors use a public dataset from previously published work [9] comprised of 22 24 h EEG recordings totaling 528 and provide the results of 22-fold leave-one-subject-out cross-validation [30]. Each of the 22 folds is a 24 h EEG recording and, therefore, consists of both the light and the dark cycle. The authors, however, propose a complex preprocessing to their single-channel EEG signal before being input to their deep learning model including an array of hand-crafted time-frequency transformations. Many existing sleep staging algorithms face challenges such as being proprietary, inadequately evaluated, dependent on complex data preprocessing, reliant on multiple EEG channels, or requiring additional signals such as EMG or EOG. Moreover, many are limited to human EEG data or trained solely on publicly available datasets. In contrast, we present an open-source, rigorously evaluated, end-to-end deep learning model trained on a novel dataset (termed Project Aurora) for sleep staging in rodents using single-channel EEG. Additionally, we contribute over 700 h of expert-scored sleep data. We compare the performance of our model with closely related methods. Furthermore, using our newly introduced rodent dataset, termed SleepyRat, we demonstrate that our model accurately predicts key sleep architecture parameters, such as vigilance state durations, bout numbers, and average bout durations.

## Methods

### Data acquisition procedure

Adult male and female rats were used across three distinct cohorts totaling 36 unique animals: 16 Wistar rats for model training and validation (termed the Project Aurora dataset), 14 Wistar rats for additional validation (termed the SleepyRat dataset), and 6 Fischer 344/Brown Norway F1 hybrid rats for additional validation with multiple experts (termed the SnoozyRat dataset). These experiments were conducted in a facility fully accredited by the Association for Assessment and Accreditation of Laboratory Animal Care (AAALAC). Rats were kept on a 12 h/12 h light-dark cycle. All protocols were approved by the Institutional Animal Care and Use Committee (IACUC) at the University of South Carolina and were in accordance with the National Institutes of Health Guide for the Care and Use of Laboratory Animals.

Rats were implanted with EEG/EMG transmitters (PhysioTel HD-S02, Data Sciences International (DSI), St. Paul, MN, USA). Briefly, under isoflurane anesthesia, rats were placed in a stereotaxic frame (Stoelting Co., Wood Dale, IL, USA). The transmitter was implanted intraperitoneally through a dorsal incision of the abdominal region. After an incision at the midline of the head was made, two EEG leads were secured to two surgical stainless-steel screws (P1 Technologies, Roanoke, VA, USA) inserted into 0.5-mm burr holes at 2.0 mm anterior/+1.5 mm lateral and 7.0 mm posterior/−1.5 mm lateral relative to bregma. The two EEG leads were secured using acrylic dental cement (Stoelting Co., Wood Dale, IL, USA). Two EMG leads were inserted into the dorsal cervical neck muscle about 1.0 mm apart and sutured into place. The skin along the head was sutured, and animals recovered for a minimum of 7 days before sleep data acquisition.

Data used in training were obtained from rats subjected to two recording sessions, each spanning 24 h [31]. Presently, the dataset is called Project Aurora dataset. Sleep data were acquired in a quiet, designated room where rats remained undisturbed for the sleep recording duration on a PC running Ponemah 6.10 software (DSI). EEG/EMG data were acquired with a sampling rate of 500 Hz low pass filtered according to Nyquist at 250 Hz by the implant. Digitized signal data were exported for both recording sessions in European Data Format (EDF). An expert in sleep staging (Project Aurora dataset was scored by Expert A) manually annotated each recording with sleep stages in 10 s epochs using NeuroScore 3.0 software (DSI). Additional details on training experts for manual sleep staging are described in Supplementary Materials.

For each of the 2 recording sessions, 16 discrete 24 h EEG/EMG signals resulted from data collection (one for each rat). We grouped the pairs of 24 h PSG recordings for each rat into 48 h PSG recordings and ensured that data was processed in a temporally consistent manner. It was critical, however, that we maintained rat identity throughout the entire investigation for proper training and evaluation of our algorithm. Therefore, we refer to the shape of the dataset as 16 rats x 48 h PSG recording, as shown in Fig. S1.

### Validation

Proper model evaluation relies on a strong validation paradigm, aiming to simulate real-world deployment [32, 33]. Machine learning models should generalize to new data, not memorize training specifics. Overfitting can occur, leading to overly optimistic results if information leaks between training and testing sets. To prevent this, we used leave-one-out cross-validation, where each rat’s data formed a unique fold. With 16 rats in the Project Aurora dataset, we trained on 15 and evaluated on the remaining one, ensuring reliable generalization, especially with limited data.

### Model

**Fig. 1:**
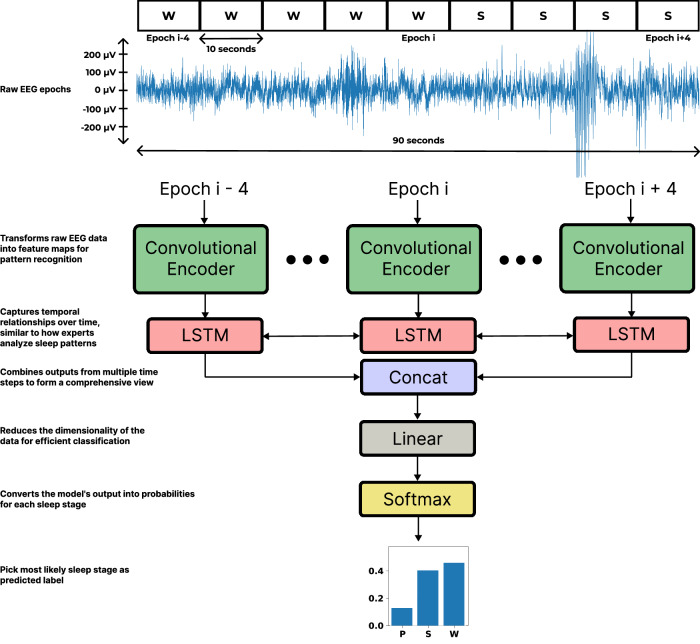
Model diagram of our algorithm. The model accepts a sequence of 9 10 s epochs to predict the class of the center 10 s epoch (epoch i, as denoted in the figure). Example raw traces are shown from our dataset to exhibit the inherent difficulty of sleep staging from a single-channel EEG. The model learns to classify the center epoch despite sometimes ambiguous patterns, a key strength of our approach. Each 10 s epoch in the sequence is independently encoded and then forwarded to a Long Short-Term Memory (LSTM) neural network to share information between neighboring 10 s epochs. The 64-dimensional LSTM output vector is linearly projected down to three dimensions and mapped to a probability distribution over 3 values (corresponding to 3 vigilant states; REM/paradoxical sleep, ‘P‘, NREM/slow-wave sleep, ‘S‘, and wake, ‘W‘) by the Softmax function.

We designed our model to first encode EEG epochs into information-rich low-dimensional vectors then incorporate sequential information from neighboring epochs to make a single sleep stage prediction. To accommodate this design, we combined a CNN [20] with a LSTM neural network [34]. We designed our model to take a sequence of 9 10 s epochs as input and predict the sleep stage of the center (5th) epoch; however, one could easily adapt the architecture to take any sequence length. Our model architecture is shown in Fig. 1.

#### Epoch encoder

First, each of the 9 10 s epochs in the input sequence is independently encoded by the epoch encoder, shown in Fig. 1. The epoch encoder is a RegNet [35], a modern variant of the CNN [20] that investigated optimal design of Residual Neural Networks [19]. The epoch encoder consists of a stack of 3 identical residual blocks [19] and a final global average pooling [36]. The number of convolution channels is shown within each residual block in Fig. S2. Each residual block consists of 3 sequences of convolution, layer normalization [37], and ReLU nonlinearity [38], as well as an elementwise residual connection before the final ReLU. The convolution kernels for each residual block are size 8, 5, and 3, in that order.

#### LSTM and classifier head

The resultant sequence of encoded feature maps is forwarded to a temporally aware neural network, resulting in a single output feature map that integrates spatial and temporal information. Lastly, this spatio-temporally-encoded feature map is presented to a final classifier that maps to 3 output states and onto a probability distribution, each of which corresponds to one of 3 vigilant states. Additional details regarding network architecture can be found in Fig. S2.

### Preparing dataset for training

We trained the model to accept an input sequence of 9 10 s epochs and output a probability distribution over 3 classes corresponding to the 3 sleep stages (REM/paradoxical sleep, ‘P’; NREM/slow-wave sleep, ‘S’; and wakefulness, ‘W’) for the epoch in the center of the sequence. To allow evaluation of the first 4 10 s epochs, we zero-padded each recording on both ends. Each zero-padded PSG signal was partitioned into 10 s epochs and transformed using a simple moving window of 9 10 s epochs with a stride of 1. We took 5% of the training set on each fold for validation.

### Optimization

We trained our model using mini-batches of size 32, where each batch consisted of sequences of 9 consecutive 10 s epochs and the corresponding sleep stage label for the center epoch. Training continued until the validation loss failed to improve for 30 consecutive epochs, implementing a regularization technique to prevent overfitting known as early stopping. This method helped ensure that we did not continue training beyond the point where model performance on unseen data began to degrade. To select the best-performing model, we conducted hyperparameter optimization using the validation set. We performed a grid search across learning rate, batch size, and sequence length to select a learning rate of 3e–4, a batch size of 32, and a sequence length of 9 epochs. This approach allowed us to systematically explore the parameter space to find an optimal configuration that balances model performance and computational efficiency.

During training, we used cross-entropy [39] as a loss function given its suitability for multi-class classification problems; it effectively measured the dissimilarity between the predicted probabilities and the true distribution of labels. For optimization, we used the Adam optimizer [40] which used adaptive learning rates for each parameter, leading to stable convergence and often better results than traditional gradient descent methods.

For reproducibility, all data processing, model training, and evaluation, were conducted in Python and PyTorch. We leveraged GPU acceleration with 2 NVIDIA RTX 4090 s.

### Evaluation metrics

Defining proper evaluation metrics is critical for establishing results [32]. Sleep staging is inherently an imbalanced classification problem. A good performance metric for evaluating an imbalanced classification is F1-score, the harmonic mean between precision and recall. In a classification setting with multiple classes, we defined the F1 Score for a given class ${C}$ as

$$\begin{array}{c}{{\mbox{F}}1{\mbox{Score}}}_{C}=\frac{2\left({{\mbox{precision}}}_{C}\right)\left({{\mbox{recall}}}_{C}\right)}{{{\mbox{precision}}}_{C}+{{\mbox{recall}}}_{C}},{\mbox{precision}}=\frac{{{\mbox{TP}}}_{C}}{{{\mbox{TP}}}_{C}+{{\mbox{FP}}}_{C}},{\mbox{recall}}=\frac{{{\mbox{TP}}}_{C}}{{{\mbox{TP}}}_{C}+{{\mbox{FN}}}_{C}}\end{array}$$

where TP was the number of true positives, FP the number of false positives, and FN the number of false negatives. We defined the macro F1 Score as the average F1 Score over each class

$$\frac{{\Sigma }_{C}{{\mbox{F}}1{\mbox{Score}}}_{C}}{N}$$

where there are ${N}$ classes.

### Comparison against previous work

In addition to testing on the Project Aurora dataset, we further tested the performance of our neural network against a recent sleep-staging algorithm published by Liu et al. [30]. Presently, the Liu et al. dataset is called the SPINDLE dataset and included data from 22 animals (mice and rats) collected across three independent sleep labs [9]. Their algorithm was based on a deep learning approach that was developed and evaluated using a publicly available dataset [9]. We utilized all available datasets from SPINDLE to evaluate our algorithm, providing a direct comparison of performance and demonstrating the generalizability of our model across species (mice and rats) and different experimental settings.

Liu et al. [30] provided results from a 22-fold leave-one-out cross-validation. The SPINDLE dataset was composed of multiple cohorts of animals sampled at varying sampling rates (128 Hz, 200 Hz, and 512 Hz) [9]. To adapt SPINDLE to our model, we resampled each EEG recording to 500 Hz using the Fourier Transform method and zero-padding (see 3.4 Data Preparation). SPINDLE provides annotations of 4 s epochs; therefore, our model’s predictions (10 s epochs) would not be directly comparable to the SPINDLE reference signal. To alleviate this problem, we resampled our prediction signal from the 10 s resolution to the 4 s resolution. Up sampling a signal from 0.1 Hz to 0.25 Hz is non-trivial since 0.1 does not divide 0.25, resulting in 1 unaligned reference 4 s epoch for every 2 predictive 10 s epochs as shown in Fig. S3. To account for this, we compared this SPINDLE reference 4 s epoch to both predictive 10 s epochs which it spans and took the mean of the metric being computed (Fig. S3A).

### Validation of conclusions on vigilance state duration and architecture with SleepyRat dataset

Data used in validating our model for conclusions on vigilance state duration and architecture were obtained from rats that were subject to discrete sleep recording sessions, as described above. Presently, the dataset is called SleepyRat dataset. An expert in sleep staging (Expert C) manually annotated each recording with sleep stages in 10 s epochs offline using NeuroScore 3.0 software (DSI) for the first 4 h of the PSG recording. All subsequent analyses for SleepyRat and SnoozyRat datasets were made between a model trained entirely on the Project Aurora dataset and transferred to be evaluated on novel datasets. Comparisons were made between LSTM scoring and the human scoring in 1 h bins from ZT 0 to 4 evaluating vigilance state duration, bout number, and average duration of each bout. Comparisons were made between LSTM scoring and human scoring using a two-way repeated measures (RM) analysis of variance (ANOVA) with ZT and scoring as within-subject factors. Post hoc analysis was conducted to evaluate changes across time with Dunnett test compared to ZT 0. All statistical analyses were performed using Prism 9.0 (GraphPad Software, La Jolla, CA, USA) and significance was defined as *P* < 0.05.

## Results

### Project aurora dataset

**Fig. 2:**
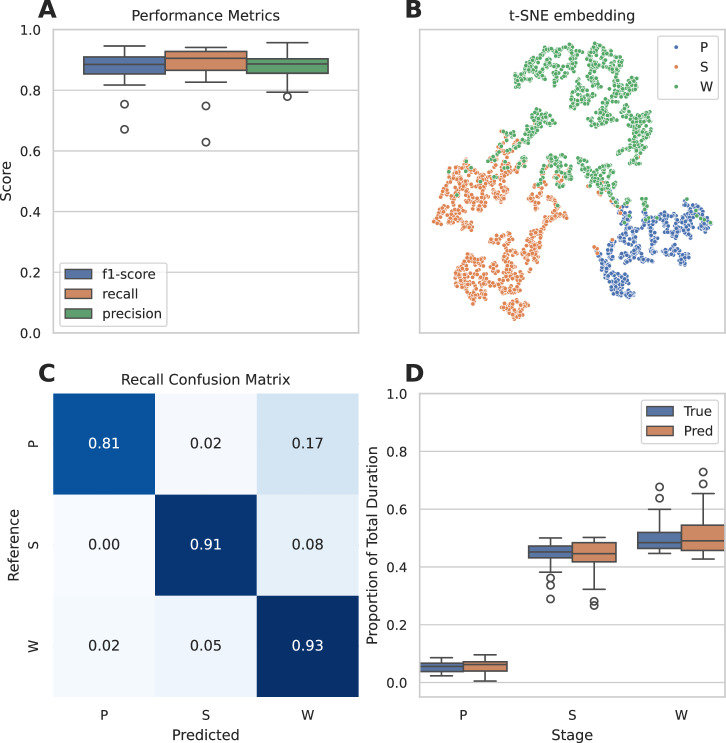
Performance of the algorithm on our Project Aurora dataset created for the model training over 16 folds of cross-validation. **A** Precision, recall, and F1-score over 16 folds of cross-validation are shown by a boxplot. Individual outliers are denoted with open circles. **B** Dimensionality reduction of EEG encodings colored by sleep stage. Our network encoded well-separated EEG signals and therefore serves as the basis for accurate sleep staging. **C** Confusion matrix. The diagonal elements represent the percentage of 10 s epochs that were correctly classified by the algorithm (recall), whereas the off-diagonal elements show the percentage of 10 s epochs mislabeled by the algorithm. **D** Duration of each sleep stage for predicted signal and reference signal over 16 folds of cross-validation expressed as a proportion of the total duration of the given EEG recording. Pairs of boxplots are shown for each stage where the left box depicts the predicted distribution, and the right box depicts the reference distribution. P – REM/paradoxical sleep, S – NREM/slow-wave sleep, W - wakefulness. Individual outliers are denoted with open circles.

The dataset consisted of 32 24 h PSG recordings obtained from 16 rats subjected to 2 recordings each. We trained our model with fixed architecture and hyperparameters over all 16 folds. The training set, for each of the 16 folds, consisted of 30 24 h EEG signals (259,200 10 s epochs). A small portion of the training set (5%) was taken to implement early stopping, a regularization technique that halts training once performance on the validation set starts to degrade. The testing set, for each of the 16 folds, consisted of 2 24 h EEG signals (17,280 10 s epochs). Fig. 2A shows a box plot displaying the performance distribution over 16 folds of cross-validation. The mean F1-score, calculated across all 16 folds, was 87.6%. One outlier incurred several misclassifications, resulting in a recall of approximately 78% and an F1-score of approximately 83% (Fig. 2A).

The model compressed 45,000-dimensional (500 Hz * 10 Seconds * 9 epochs) raw EEG inputs into a 64-dimensional latent representation before being linearly projected into three dimensions for sleep staging. To visualize similarities between these 64-dimensional feature maps, we show the results of tSNE [41], a dimension reduction algorithm designed to preserve high-dimensional neighborhoods, in Fig. 2B. NREM/slow-wave sleep, ‘S’, and wakefulness, ‘W’, stages were well-clustered whereas many wake and REM/paradoxical sleep, ‘P’, stages’ embeddings escaped into the other’s neighborhood. The similarity between EEG waveforms during wakefulness and REM/paradoxical sleep almost certainly contributed to these mixtures.

We tested the sleep staging performance of individual vigilance states (REM/paradoxical sleep, NREM/slow-wave sleep, and wakefulness) by computing a recall confusion matrix (Fig. 2C). The average recall for REM/paradoxical sleep was 81.1%, and most misclassifications were classified as wakefulness. Both NREM/slow-wave sleep and wakefulness had a recall over 91.2%. Class specific recall, precision, and F1 score can be found in Table 1.

**Table 1:** Performance of our model on our Project Aurora dataset broken down by class.

Class	F1	recall	precision
P	78.2 ± 14.0	81.1 ± 16.6	78.5 ± 12.5
S	92.3 ± 5.0	91.2 ± 9.4	94.1 ± 3.8
W	92.2 ± 2.9	93.1 ± 3.8	91.8 ± 6.4

Metrics are the mean and standard deviation over 16 folds of cross-validation for F1-score, recall, and precision.

The dynamics of the composition of sleep, in terms of sleep stages, are complex but important for interpretations of sleep quality in preclinical studies. A few important parameters of sleep composition can thus serve as a unique characteristic for evaluation of model performance and include total bout duration, average bout duration, and number of bouts. We investigated these parameters (Fig. S4) and show the total duration of each stage as a proportion of EEG recording time in Fig. 2D.

Tezuka et al. [29] report a mean F1-score of 80.8 across 10 folds of cross-validation, which is notably lower than the F1-score achieved by our model. Consequently, we focused our comparison on SPINDLE, where the performance gap is more meaningful for further evaluation.

### SPINDLE dataset

It is critical to emphasize that our model was optimized for sleep staging with 10 s epochs and that stages in the SPINDLE dataset were scored with 4 s epochs. In evaluating our model on the SPINDLE dataset, we found that, even by unique characteristic of 10 s epochs, we outperformed the algorithm proposed by Liu et al. [30] despite theirs being optimized specifically for the SPINDLE dataset. The results of the 22-fold leave-one-out cross-validation on the SPINDLE dataset are reported in Table S1. Notably, using the same dataset and validation scheme, our model achieved a mean F1-score of 89.6% compared to 88.1% in Liu et al. [30]. Though we achieved higher performance in all three classes, most of the performance increase came from the REM/paradoxical sleep class where our model achieved F1-score of 83.6% as opposed to 79.4% in Liu et al. [30]. We emphasize that our results in Table S1 reflect our performance after up sampling our model’s 10 s prediction signal to a 4 s prediction signal. We performed further analysis on the distribution of performance across the 22 folds of cross-validation, the results of which are shown in Fig. S5.

Notably, one outlier achieved an F1-score of approximately 83%. To further understand model performance broken down by class, we present the mean recall confusion matrix over the 22 folds of cross-validation (Fig. S5C). Most misclassifications occurred between REM/paradoxical sleep and wakefulness. For each sample, our model outputs a probability distribution over all possible stages; therefore, we can quantify the model’s confidence in predicting a certain class. For each fold, we took the average confidence of the model and compared this to the macro F1-score for that recording (Fig. S5B). Linear regression between these two variables suggested a positive relationship between the model’s average confidence and the model’s performance. We investigated the proportion of recording time that each stage constituted as well as the proportion of total EEG recording time predicted by the model (Fig. S5D). We performed linear regression between the SPINDLE reference values and predicted values for all combinations of classes and all three parameters (Fig. S4). Strong positive linear correlation was confirmed for all combinations of classes and parameters. A common method of visualizing sleep stages for a given EEG recording is a hypnogram (a graph with a time-based domain and a discrete range typically consisting of states of vigilance or sleep stages). The reference hypnogram is shown in Fig. S6A and our model’s predicted hypnogram is shown in Fig. S6B. Further, the EEG signal that constitutes the input to the model is shown in Fig. S6C. Lastly, the predicted probability distribution over all stages by the model for the EEG signal is shown in Fig. S6D. We took the highest probability as the predicted class, and the minimum confidence with which our model could predict a certain class was slightly above 0.33. The region from epoch 150 to epoch 250 on Fig. S6D shows where the network was almost 100% confident.

### SnoozyRat dataset

SnoozyRat dataset was conducted in a cohort of rats to evaluate our novel sleep scoring network against two separate expert scorers (Expert A and Expert B). Correlational analysis confirmed robustness of our sleep scoring network, evaluating vigilance state durations, bout numbers, and average bout duration across 24 h. Evaluation of the data across 24 h provides support for the accuracy of the sleep scoring network across light-dark phases. Importantly, we demonstrate significant correlation between Expert A and Expert B scoring. Data are shown in Figs S7, S8, S9, and S10.

### SleepyRat dataset

**Fig. 3:**
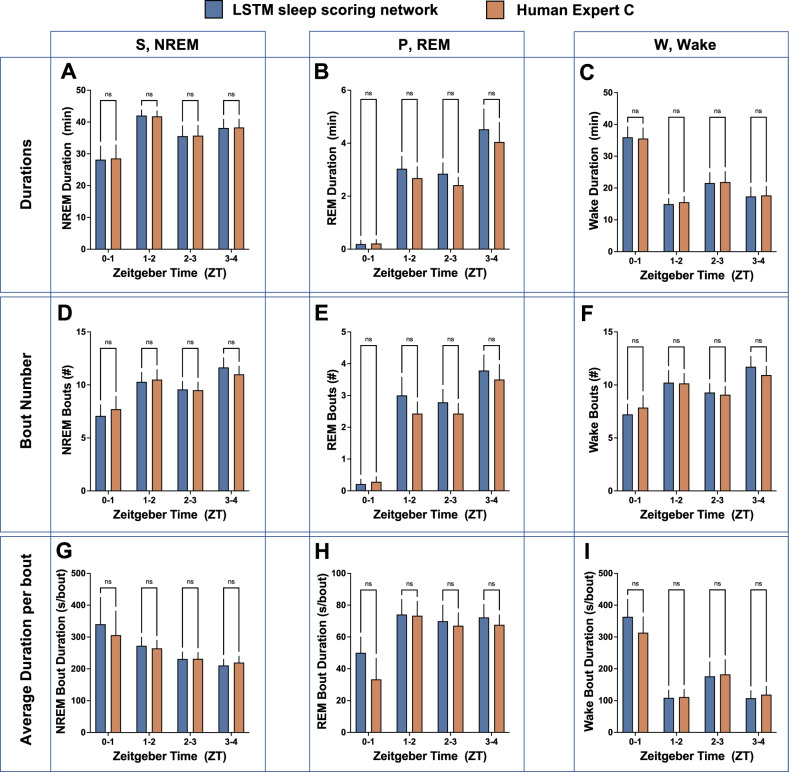
Sleep-wake duration and architecture data do not significantly differ between LSTM sleep scoring network and human expert scoring of SleepyRat dataset. **A** NREM duration, **B** REM duration, **C** Wake duration, **D** NREM bout number, **E** REM bout number, **F** Wake bout number, **G** average NREM bout duration, **H** average REM bout duration, **I** average Wake bout duration. Two-way RM ANOVA with post-hoc Šídák’s multiple comparisons test. ns not significant. Data are mean ± SEM. N = 14 per group.

The SleepyRat dataset was conducted in a cohort of rats to evaluate our novel sleep scoring network. We evaluated vigilance state durations and architecture comparing the findings of LSTM scored data to an expert human (Expert C; a different expert from Project Aurora and SnoozyRat datasets). We presently focused on evaluating the data in 1-hr bins during the early light phase, wherein rats typically accumulate the largest proportion of sleep. We selected the SleepyRat dataset for our validation because the animals were handled by an experimenter at ZT0, thereby their transition to sleep was disrupted at the start of the light phase. As can be seen in Fig. 3, the REM sleep duration was on average less than a minute during the first hour (ZT 0–1) due to the experimental handling. However, in the subsequent hours of our analysis, sleep rebound occurred, wherein a significant increase in NREM sleep and REM sleep duration is noted by both the LSTM sleep scoring network and Expert C human scoring. Importantly, we determined no significant differences in the data due to scoring (Table 2 and Fig. 3), but found significant impact of time of day, ZT, on vigilance state duration and architecture (bout number and average duration of vigilance bouts) parameters. Post-hoc Šídák’s multiple comparisons test revealed significant differences between ZT 0–1 and subsequent time bins for parameters showing a main effect of ZT. Notably, LSTM and Expert C scoring showed consistent findings on the impact of time on vigilance state duration and architecture parameters (Fig. 3). Additionally, as shown in Fig. S11, a separate analysis using a two-way RM ANOVA with post-hoc Dunnett test confirmed no significant differences between LSTM and human scoring for these parameters, further validating the robustness of our LSTM scoring network.

**Table 2:** Results of SleepyRat dataset statistical analysis comparing LSTM scoring and human Expert C scoring.

Vigilance State	Scoring	ZT	Interaction
Duration			
S, NREM	0.4852	0.0423^a^	0.3153
P, REM	0.1281	<0.0001^a^	0.2553
W, Wake	0.3713	0.0008^a^	0.2074
Bout Number			
S, NREM	0.9327	0.0341^a^	0.0516
P, REM	0.1857	<0.0001^a^	0.4208
W, Wake	0.8179	0.0535	0.0876
Average Bout Duration			
S, NREM	0.4614	0.1865	0.2871
P, REM	0.3060	0.4495	0.3416
W, Wake	0.4412	0.0023^a^	0.0624

P-values are indicated for main effects of scoring, ZT, and interaction between scoring and ZT.

^a^indicates significant effect wherein *P *< 0.05. P-values of two-way RM ANOVA.

## Discussion

The gold standard for sleep staging involves a trained human expert manually annotating PSG, typically using EEG and EMG signals, to classify sleep stages. Developing a reliable, automated algorithm for sleep staging is critical for improving the efficiency of EEG signal processing. An automated method that uses only single-channel EEG recordings would provide a significant advancement by reducing the need for multi-channel recordings and manual annotations.

Human experts, after extensive training, rely on visual inspection of signals (such as EEG and EMG) and feature-based tools (like power-spectral density) to classify sleep epochs into vigilant stages. The patterns within these signals (e.g., amplitude and frequency) ultimately guide expert classifications. To replicate this process, we implemented a CNN to encode visual patterns contained in raw EEG signals down to a representation more amenable to classification. Each 10 s epoch is processed independently, resulting in a set of feature maps. To train and validate this network, we conducted extensive training and evaluation using the Project Aurora dataset. This dataset consists of over 700 h of single-channel EEG recordings from a cohort of 16 rats recorded across two 24 h sleep sessions.

To capture temporal relationships between EEG patterns, we introduced a “temporal encoder” following the spatial encoder. This temporal encoder was designed to share information across time by considering neighboring epochs centered around the target epoch to be classified. This mirrors the 90 s window used by human scorers when analyzing PSG. The temporal encoder is based on an LSTM network, which is capable of learning and integrating temporal dependencies from the data. The combination of spatial and temporal encoders allows our model to produce an intermediate representation rich in spatiotemporal information.

Not only does our model effectively capture spatiotemporal information, but it also ensures that the representations of different sleep stages are well-separated in the latent space, which is key for accurate classification. This capability of deep learning—to automatically transform raw data into meaningful representations for downstream tasks—highlights the strength of our approach. Our end-to-end model takes raw single-channel EEG signals as input and outputs sleep stage classifications, avoiding the need for feature extraction steps that could introduce bias or redundancy.

While true labels are required for direct accuracy assessment, our model’s outputs, including epoch encodings and predictions, are directly available for plotting in any preferred statistical software. This means that researchers can easily export these outputs to visualize them in software like R, Python (with libraries like Matplotlib or Seaborn), or MATLAB. Such visualization aids in understanding how our model behaves with different datasets, offering a practical approach for users to engage with our model’s results without needing to compute true labels.

To further validate the usability of our model, we conducted an extensive evaluation using the SleepyRat dataset. This dataset consists of over 224 h of single-channel EEG recordings from a cohort of 14 rats recorded across four 4 h sessions. The dataset provides a robust foundation for evaluating the model’s performance in terms of vigilance state durations, bout numbers, and the average duration of bouts. A two-way repeated measures ANOVA was performed to compare the LSTM-based sleep staging results with human expert scoring. No significant differences were found between the LSTM predictions and human annotations for sleep stage durations and architecture parameters, confirming the model’s reliability for preclinical sleep analysis. As shown in Fig. 3, the consistency in the number of bouts and the average bout durations across different sleep stages between LSTM scoring and human scoring further validates the model’s effectiveness.

Additionally, we provide comparisons to existing datasets like SPINDLE, which consisted of 22 folds and under 500 h of EEG recordings. Our model was rigorously evaluated using leave-one-out cross-validation on both the newly introduced SleepyRat dataset and the SPINDLE dataset. In total, 38 deep learning models were developed during the validation phase, and performance was measured using F1 score, precision, and recall.

Through the introduction of the SleepyRat dataset and the validation performed using ANOVA, we demonstrated that our deep learning model automates sleep staging effectively and provides reliable estimates of sleep architecture parameters. This validation, combined with the model’s performance on the Project Aurora dataset, ensures usability for large-scale preclinical sleep analysis and offers a significant reduction in the time and resources required for manual sleep staging.

### Citation diversity statement

The authors have attested that they made efforts to be mindful of diversity in selecting the citations used in this article.

## Supplementary information

Supplementary Material

## Data Availability

The data presented in this study are available on request from the corresponding author. The SPINDLE dataset can be found at https://sleeplearning.ethz.ch/paper/. The source code and documentation of the algorithm are available at https://github.com/smithandrewk/sleepyrats-ml. The Python code to reproduce all the results and figures of this paper can be found at https://github.com/smithandrewk/sleepyrats-ml. All analyses were conducted in Python 3.11 using PyTorch 1.13.1.

## Code availability

The data presented in this study are available on request from the corresponding author. The SPINDLE dataset can be found at https://sleeplearning.ethz.ch/paper/. The source code and documentation of the algorithm are available at https://github.com/smithandrewk/sleepyrats-ml. The Python code to reproduce all the results and figures of this paper can be found at https://github.com/smithandrewk/sleepyrats-ml. All analyses were conducted in Python 3.11 using PyTorch 1.13.1.
