# Supplementary material for: A deep learning software tool for automated sleep staging in rats via single channel EEG
Source: NPP Digit Psychiatry Neurosci. 2025 Jul 10;3:20. doi: 10.1038/s44277-025-00035-y (PMC12245713; doi:10.1038/s44277-025-00035-y)
Supplement: Supplementary file 1 — Supplementary Material [file 44277_2025_35_MOESM1_ESM.docx]

*Supplementary Material*

A deep learning software tool for automated sleep staging in rats via single channel EEG

Andrew Smith^1^, Snezana Milosavljevic^2^, Courtney Wright^2^, Charlie Grant^2^, Ana Pocivavsek^2^, Homayoun Valafar^1*^

^1^Computer Science and Engineering, University of South Carolina, Columbia, 29208, SC, USA.

^2^Department of Pharmacology, Physiology, and Neuroscience, University of South Carolina School of Medicine, Columbia, 29208, SC, USA.


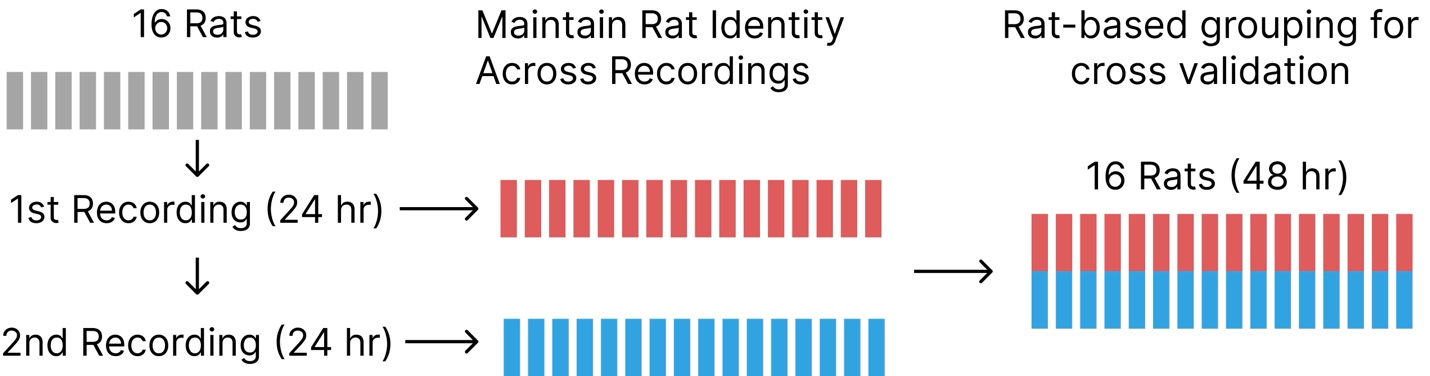


**Figure S1. Diagram of dataset creation.** PSG data acquired for 24 hours for each of 16 rats producing 16 discrete 24-hour PSG recordings. The same 16 rats underwent PSG data acquisition for another 24 hours producing another set of 16 discrete 24-hour PSG recordings. Rat identity was maintained across these two recording sessions. We grouped both sets of PSG recordings across the rats for proper algorithm training. The grouped set of PSG recordings is shown on the right with 16 vertical rectangles each representing 48 hours of PSG data. We refer to the dataset as 16 48-hour PSG recordings for simplicity.


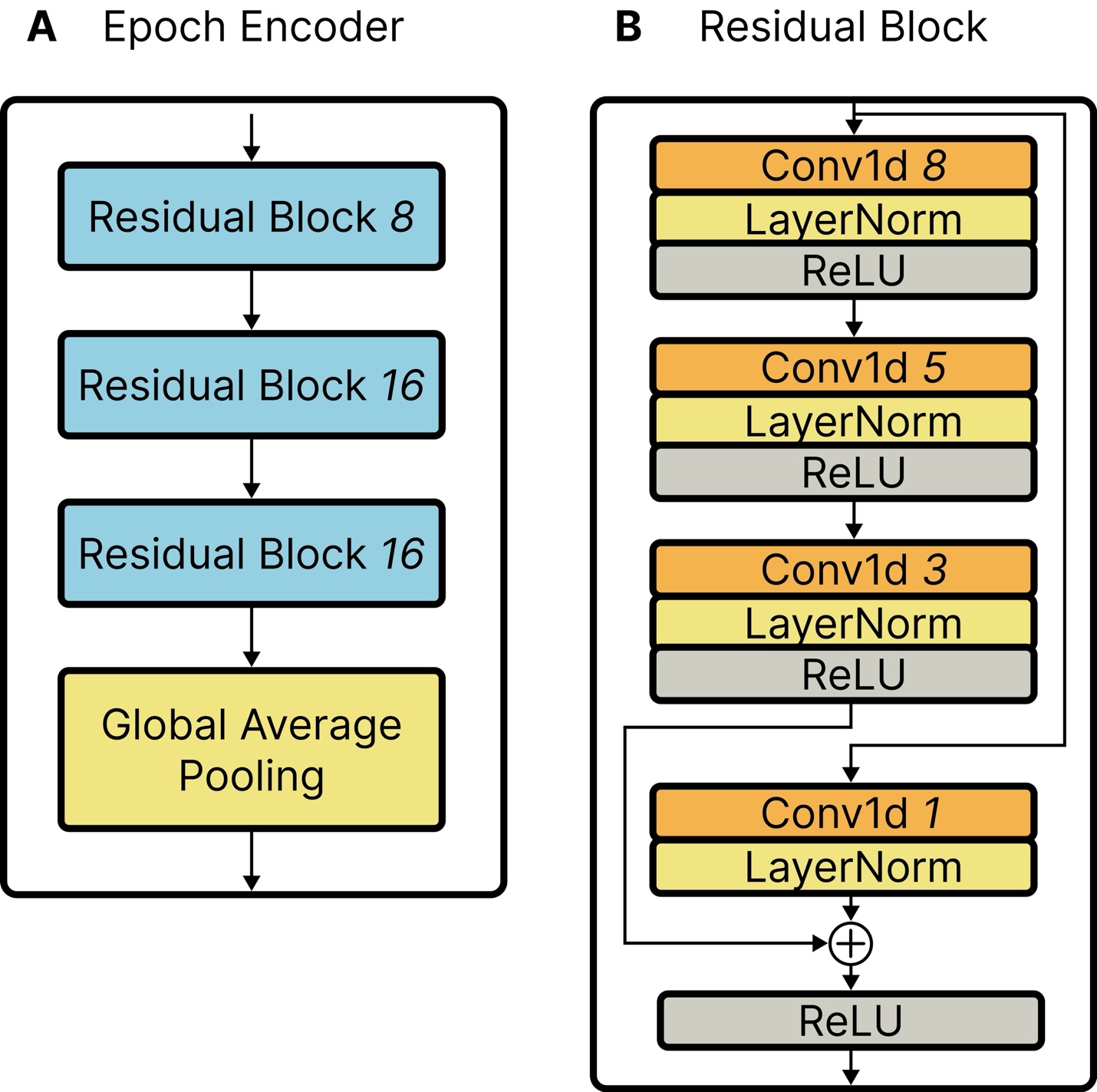


**Figure S2. Diagram of fundamental components of our algorithm.** (A) The 10-second epoch encoder, and (B) The residual block. The 10-second epoch encoder consists of a sequence of residual blocks and then global average pooling. Each residual block has a number that denotes the number of latent feature maps to use in that block. The residual block consists of a sequence of convolutions, normalization, and nonlinearities. There is a residual summation before the final nonlinearity. Conv1d layers have a number that denotes the kernel size of convolution.


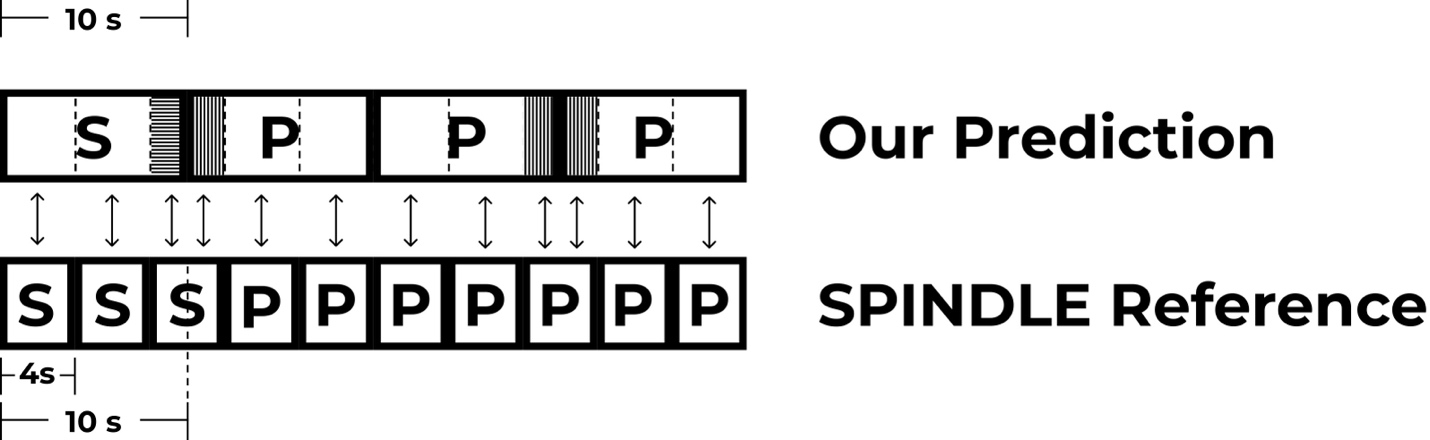


**Figure S3. Comparison between sleep stages in our prediction signal and the SPINDLE reference signal.** Our network predicts sleep stages in 10-second epochs, but the SPINDLE dataset provides a reference in 4-second epochs. Therefore, we devised a reasonable method of comparison shown here. Without loss of generality, we represent two different sleep stages (they may be any two) by vertical lines and horizontal lines, respectively. For each reference 4-second epoch, the epoch is either contained within our prediction 10-second epoch or evenly spans two of our prediction 10-second epochs. For each reference 4-second epoch entirely contained within a prediction 10-second epoch, we compare these sleep stages directly. For the reference 4-second epoch that evenly spans two of our prediction 10-second epochs, we evaluate average correctness over the two prediction 10-second epochs. (A) If an S reference 4-second epoch spans an S-P pair of prediction 10-second epochs, we assign an accuracy of 0.5. (B) If a P reference 4-seconds epoch spans a P-P pair of prediction 10-second epochs, we assign an accuracy of 1. (C) If the prediction 10-second epochs are both incorrect, we assign an accuracy of 0. S – NREM/slow-wave sleep, P – REM/paradoxical sleep.


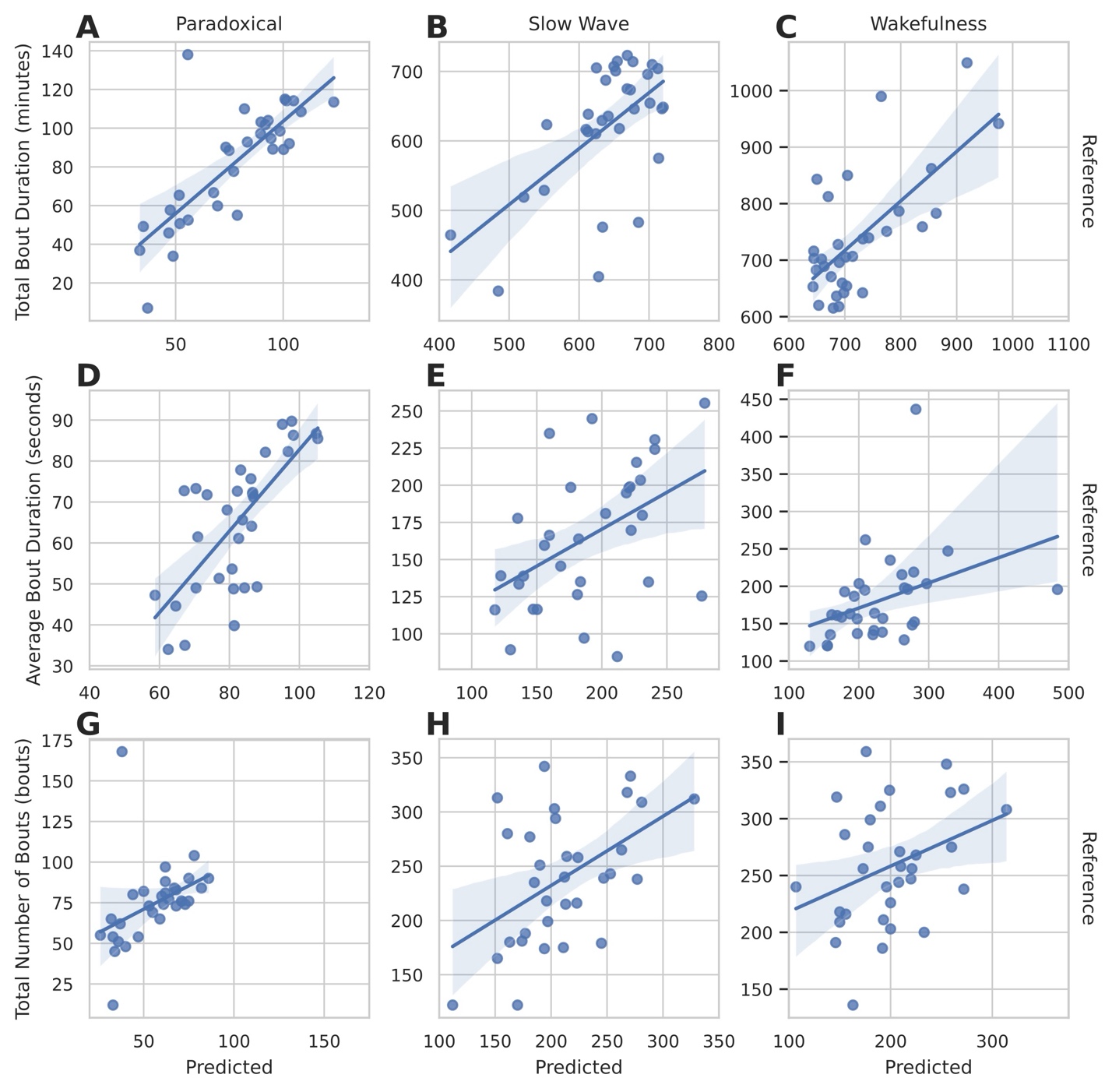


**Figure S4. Performance of our algorithm on parameters of the testing set.** Panels (A-C) show the total bout duration for REM/Paradoxical Sleep, NREM/Slow Wave Sleep, and Wakefulness, respectively, against predicted values. Panels (D-F) display the average bout duration for REM/Paradoxical Sleep, NREM/Slow Wave Sleep, and Wakefulness, respectively, against predicted values. Panels (G-I) present the total number of bouts for REM/Paradoxical Sleep, NREM/Slow Wave Sleep, and Wakefulness, respectively, against predicted values. Linear regression was applied for all combinations of sleep stages and parameters. The shading represents the 95% confidence interval, and the x-axis shows the predicted values while the y-axis shows the measured (or reference) values. The analysis demonstrates the model’s ability to predict bout parameters with reasonable confidence, with tighter fits in some stages and parameters compared to others.


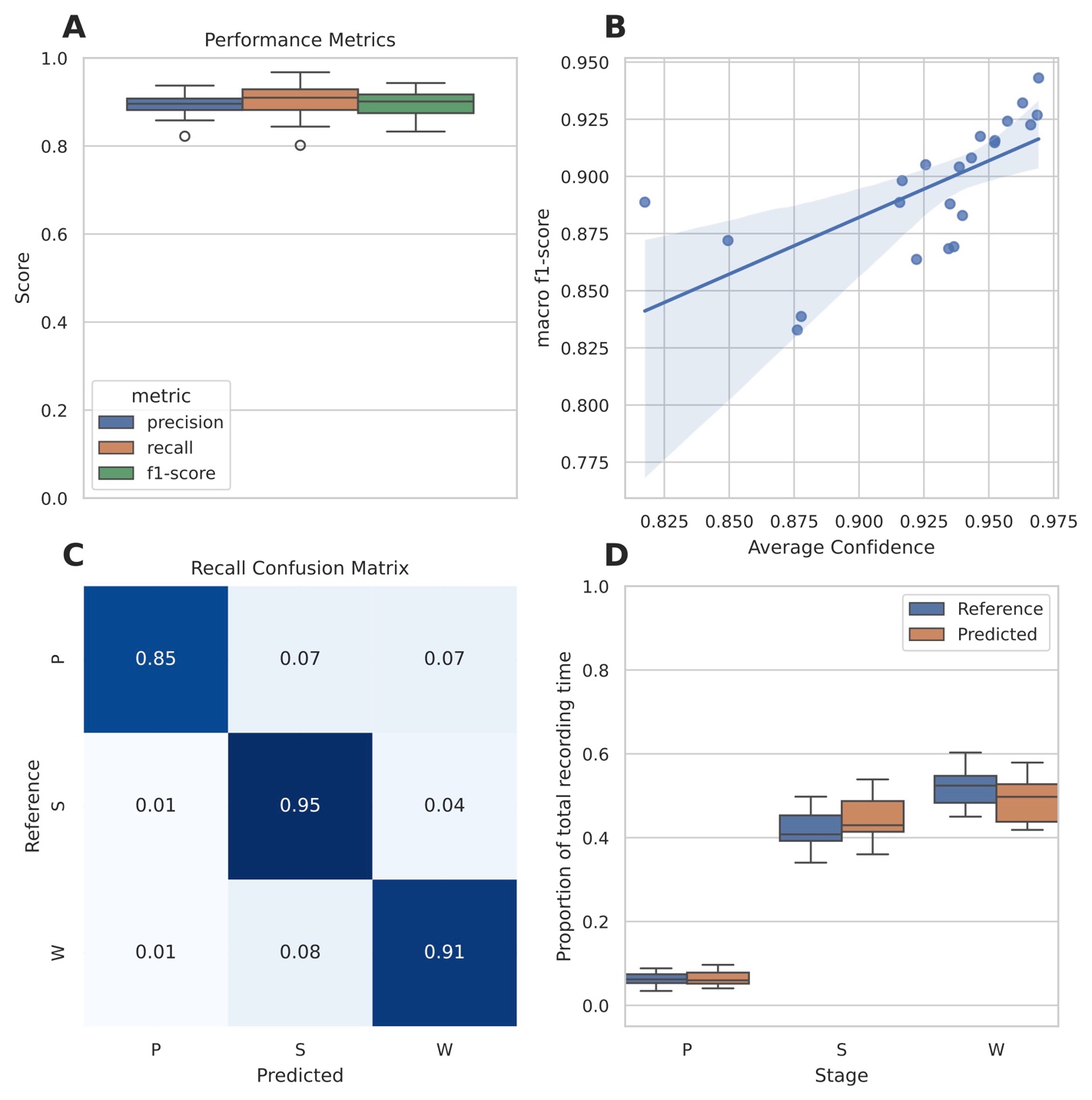


**Figure S5. Performance of the algorithm on the SPINDLE dataset over 22 folds of cross-validation** (A) Precision, recall, and f1-score over 22 folds of cross-validation are shown by a boxplot. (B) Correlation between macro f1-score and average confidence levels of the algorithm. Overall confidence was calculated for each EEG recording by averaging the confidence levels across all 10-second epochs. (C) Confusion matrix. The diagonal elements represent the percentage of 10-second epochs that were correctly classified by the algorithm (recall), whereas the off-diagonal elements show the percentage of 10-second epochs mislabeled by the algorithm. (D) Duration of each sleep stage for predicted signal and reference signal over 16 folds of cross-validation expressed as a proportion of the total duration of the given EEG recording. Pairs of boxplots are shown for each stage where the left box depicts the predicted distribution, and the right box depicts the reference distribution. P – REM/paradoxical sleep, S – NREM/slow wave sleep, W - wakefulness.


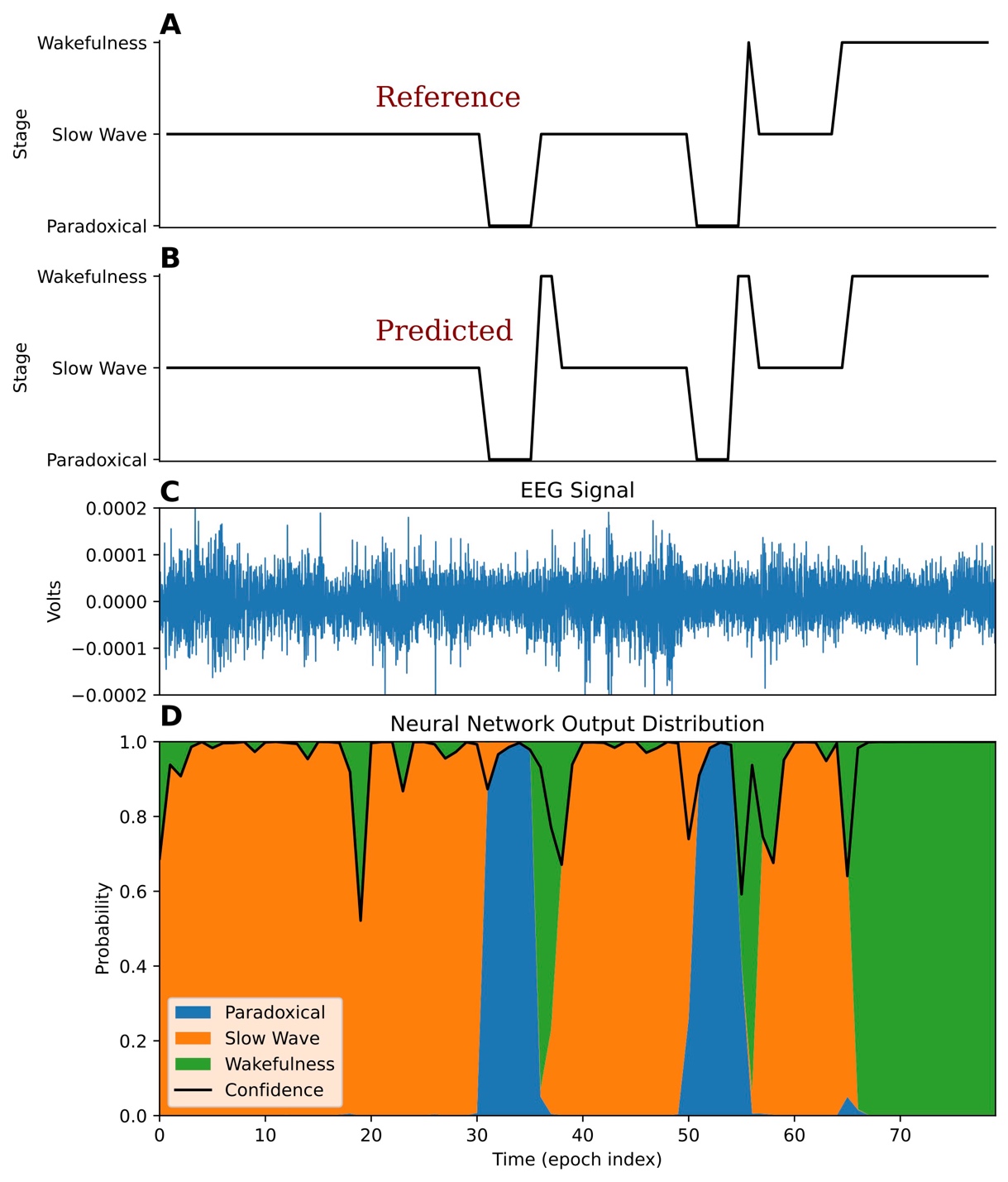


**Figure S6. Stacked representation of algorithm predictions, raw data, and algorithm confidence for one fold.** (A) The reference hypnogram as annotated by a human expert. (B) The predicted hypnogram as predicted by the algorithm. (C) The raw EEG signal that is used as the sole network input. (D) The probability distribution over sleep stages produced by the network. Additionally, the confidence level of the network is denoted by black line. P – REM/paradoxical sleep, S – NREM/slow wave sleep, W - wakefulness.


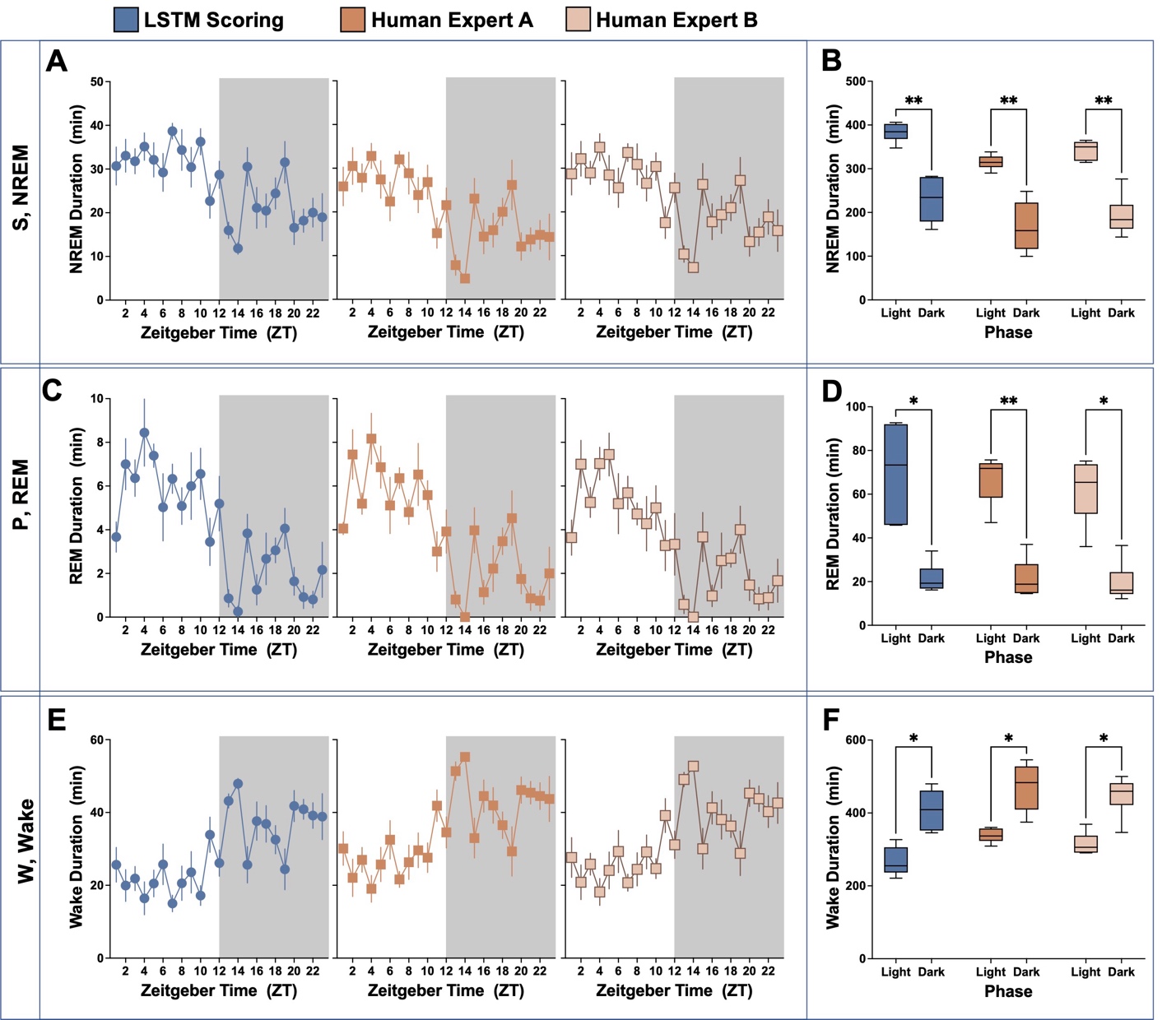


**Figure S7. Concordance of sleep-wake classification of SnoozyRat dataset by LSTM scoring and two human experts.** Vigilance state durations were evaluated in 1-hr bins during the light phase (Zeitgeber Time (ZT) 0 – 12) and dark phase (ZT 12-24). LSTM and human experts classified more NREM and REM sleep during the light phase and more wake during the dark phase. (A) NREM sleep duration in 1- hr bins. (B) Total NREM sleep duration by phase. (C) REM sleep duration in 1-hr bins. (D) Total REM sleep duration by phase. (E) Wake duration in 1-hr bins. (F) Total Wake duration by phase. RM ANOVA with Bonferroni’s post-hoc test. * P < 0.05, **P < 0.01. Data are mean ± SEM. N=6/group.


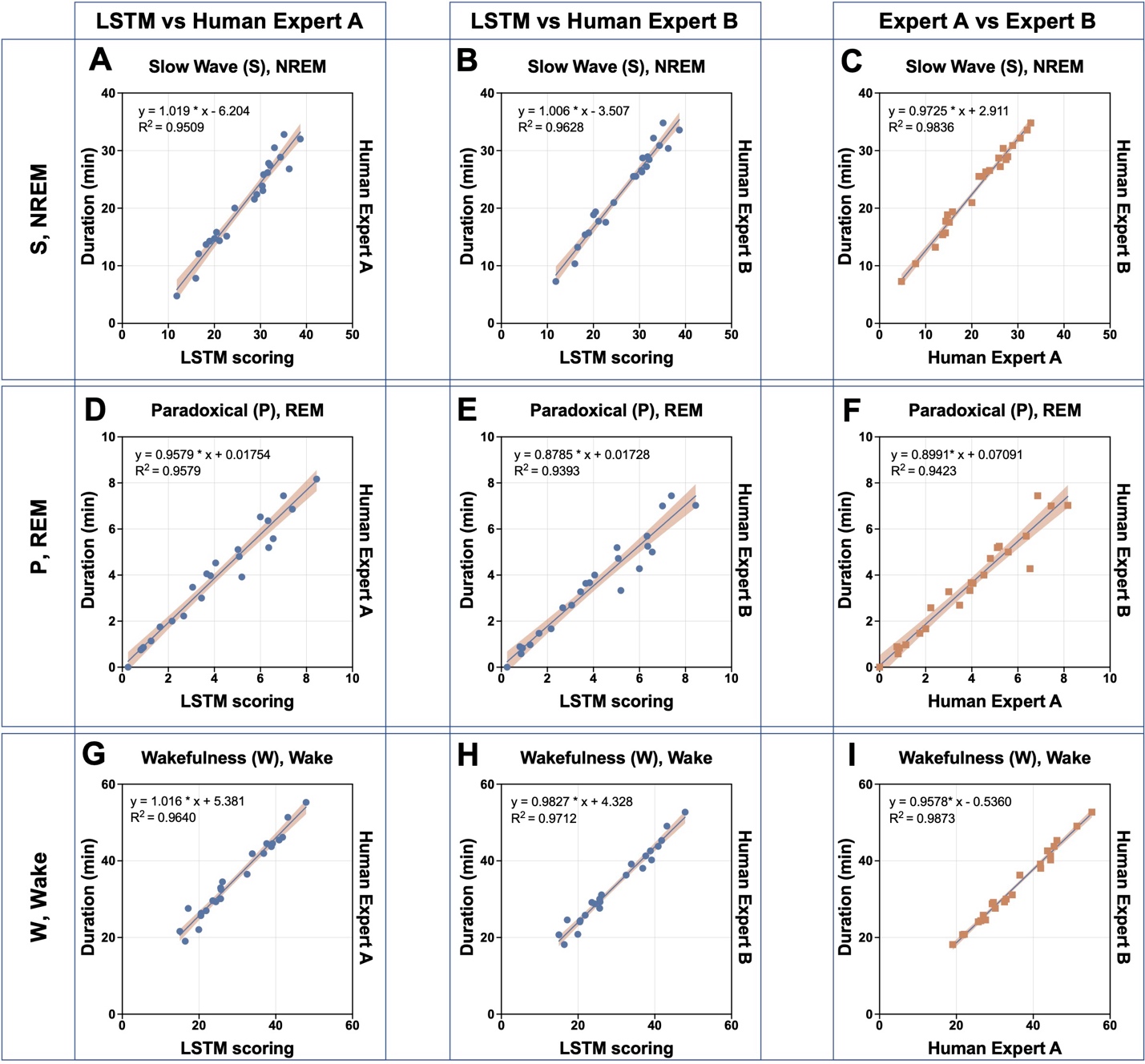


***Figure S8. Significant correlations of vigilance state durations across 24-hours:*** ***LSTM Scoring vs. Human experts and inter-rater agreement among experts****. Correlation analysis was conducted with SnoozyRat dataset to evaluate vigilance state durations between LSTM scoring and human experts, as well as among human experts. (A-C) NREM sleep duration. (D-F) REM sleep durations. (G-I) Wake duration. Linear regression was applied for all analyses and R^2^ greater than 0.94 for all analyses demonstrated the LSTM model’s ability to predict vigilance state durations and high inter-rater agreement between human experts.*


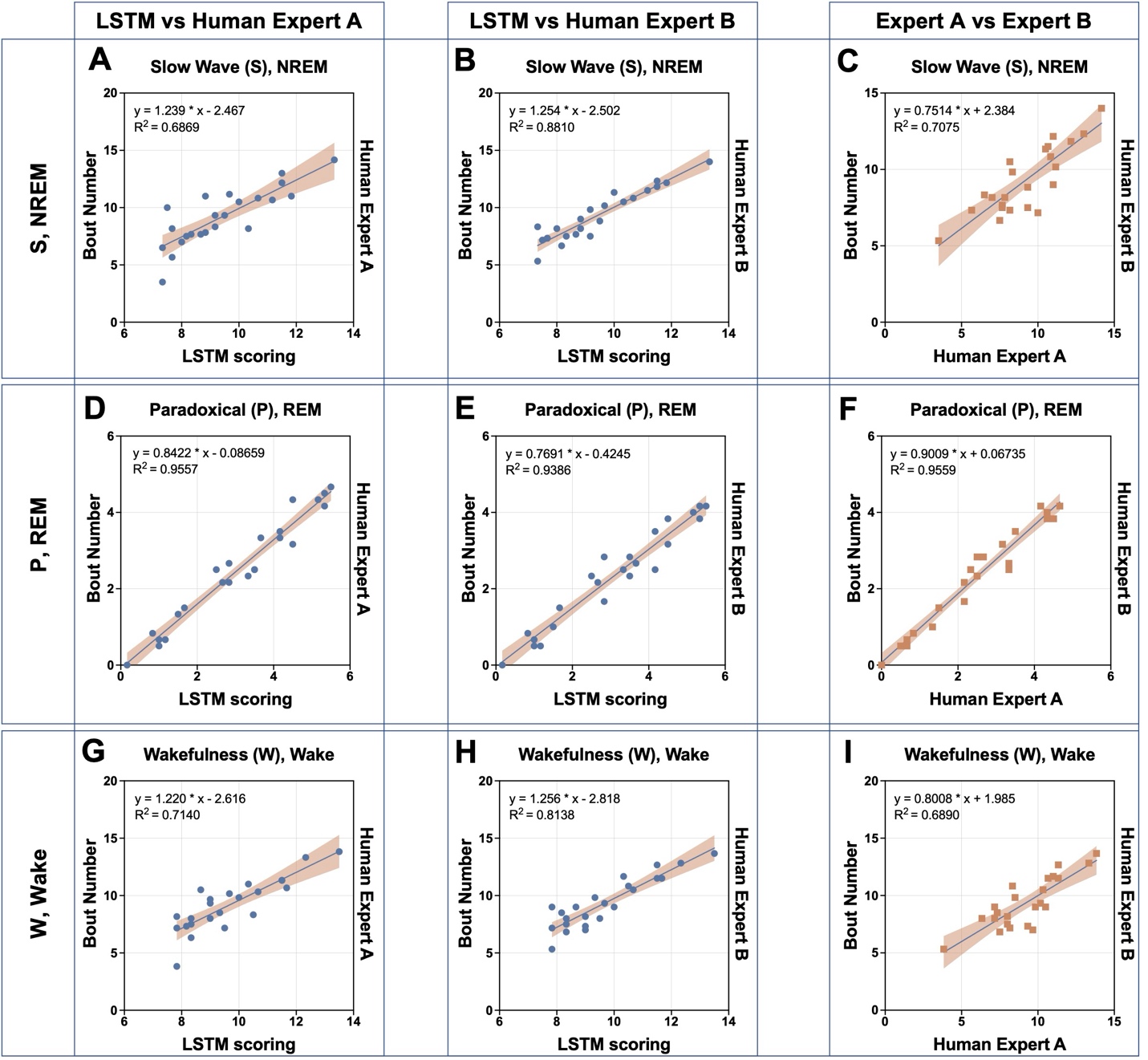


***Figure S9. Significant correlations of vigilance state bout numbers across 24-hours:*** ***LSTM Scoring vs. Human experts and inter-rater agreement among experts****. Correlation analysis was conducted with SnoozyRat dataset to evaluate vigilance state bout numbers between LSTM scoring and human experts, as well as among human experts. (A-C) NREM sleep bouts. (D-F) REM sleep bouts. (G-I) Wake bouts. Linear regression was applied for all analyses and demonstrated the LSTM model’s ability to predict vigilance state bout numbers and the inter-rater agreement between human experts.*


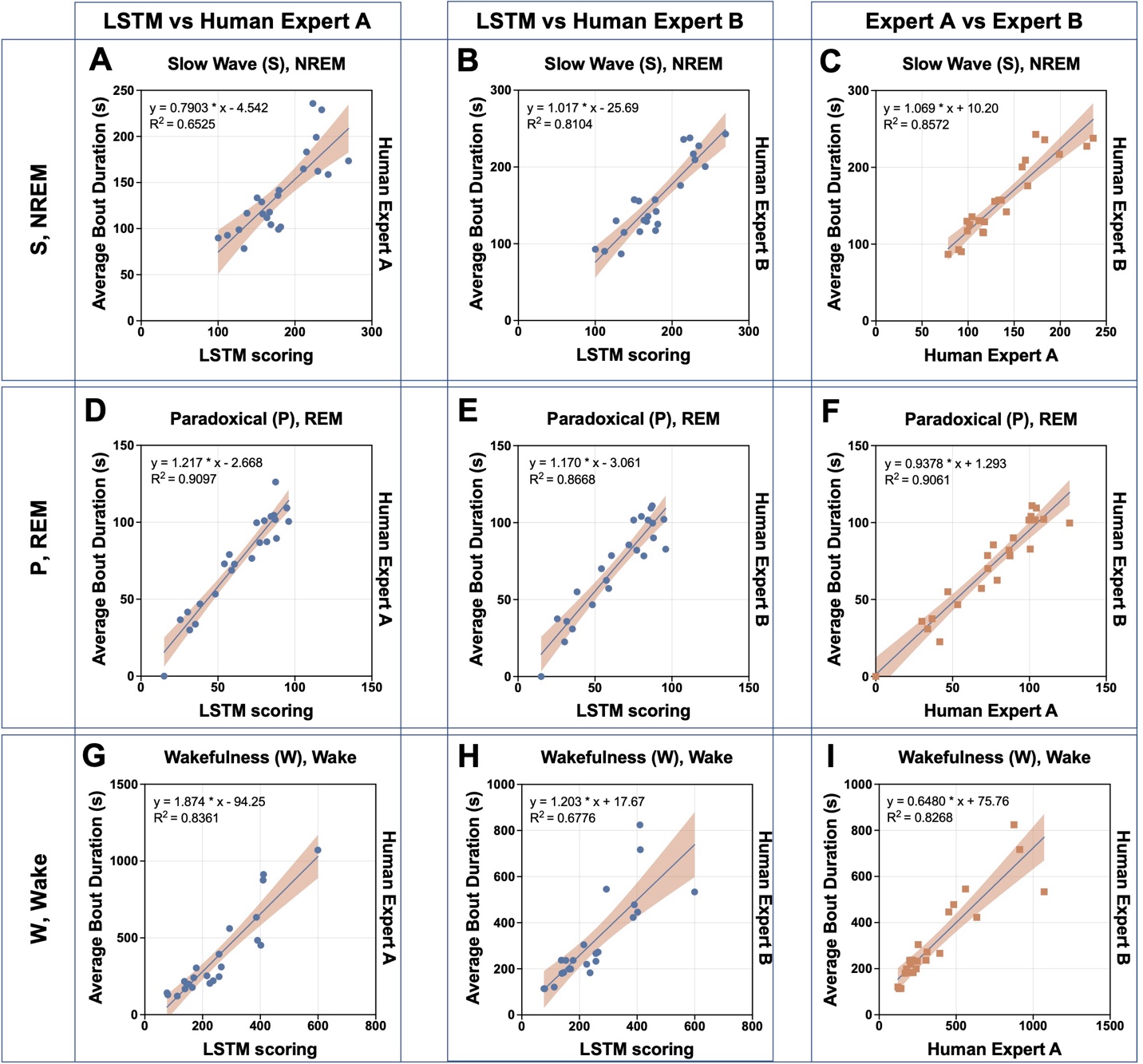


***Figure S10. Significant correlations of vigilance state average bout durations across 24-hours:*** ***LSTM Scoring vs. Human experts and inter-rater agreement among experts****. Correlation analysis was conducted with SnoozyRat dataset to evaluate vigilance state average bout durations between LSTM scoring and human experts, as well as among human experts. (A-C) NREM sleep average bout durations. (D-F) REM sleep average bout durations. (G-I) Wake average bout durations. Linear regression was applied for all analyses and demonstrated the LSTM model’s ability to predict vigilance state bout numbers and the inter-rater agreement between human experts.*


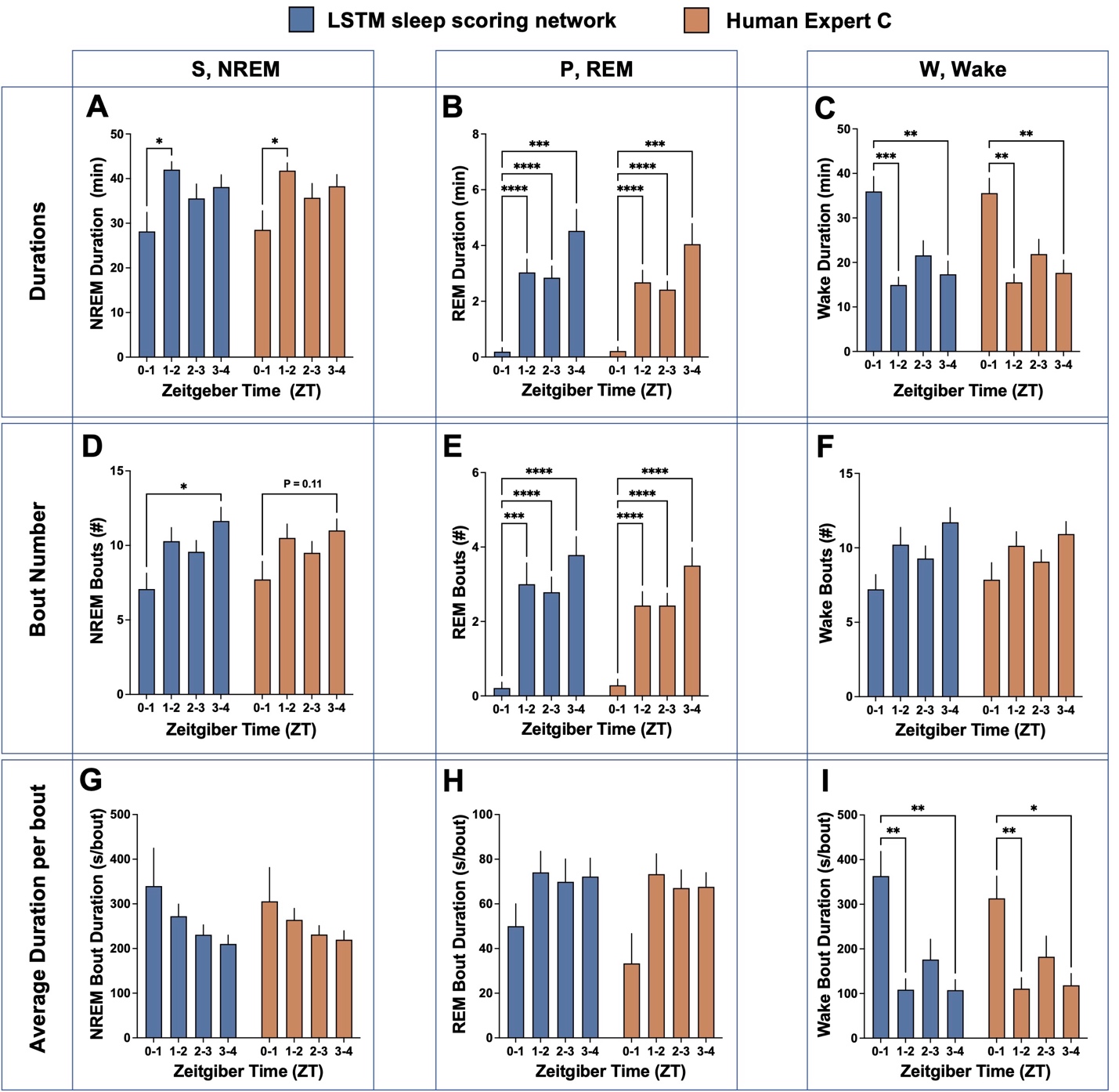


**Figure S11: Sleep-wake duration and architecture data do not significantly differ between LSTM and human expert scoring of SleepyRat dataset.** (A) NREM duration, (B) REM duration, (C) Wake duration, (D) NREM bout number, (E) REM bout number, (F) Wake bout number, (G) average NREM bout duration, (H) average REM bout duration, (I) average Wake bout duration. Two-way RM ANOVA with post-hoc Dunnett test. * P < 0.05, ** P < 0.01, *** P < 0.001, **** P < 0.0001. Data are mean ± SEM. N =14 per group.

**Table S1. Performance of our model and Liu et. al model on SPINDLE dataset.** Metrics are the mean of 22 folds of cross-validation. Data are reported as mean ± standard deviation where applicable, otherwise, just the mean value is reported.

| Metric | Ours (4s) | Liu et. al, 2021 (4s) |
| --- | --- | --- |
| Precision | **89.4 ±** 2.6 | 87.1 |
| Recall | **90.2 ±** 3.9 | 89.2 |
| F1 | **89.6 ±** 2.9 | 88.1 |
| F1 P | **83.6** | 79.4 |
| F1 S | 92.1 | **92.5** |
| F1 W | **93.0** | 92.4 |

# Training Experts for Manual Sleep Staging

# Experienced experts teach new sleep scorers (trainees) to score sleep using the following guidelines. Trainees are deemed experts once they have completed the training and independently scored a new cohort of sleep recordings.

# Workspace Settings: The scoring window display spans 60-90s (6-9, 10-s epochs) on a wide-screen computer monitor. A scorer will display EEG (-0.5 mV to +0.5 mV), EMG (-0.5 mV to +0.5 mV), activity (0 to 5 counts), and power bands.

# Scoring Criteria: Sleep states are scored as wake (low-amplitude high-frequency EEG, high amplitude EMG, peaks of activity count), NREM sleep (high-amplitude low-frequency EEG, minimal EMG, no activity counts), or REM sleep (low-amplitude high-frequency EEG, absent EMG, no activity counts, high theta (4-7 Hz) power relative to delta (0-4 Hz) power). Transitions from wake to NREM, NREM to REM, NREM to wake, and REM to wake are allowed. Transitions are typically marked if a change in a sleep state is constant for at least two epochs (>20s).

# Training Procedure: An expert reviews the above information and provides the trainee with 2-3 previously scored 24-hour recordings. It is important that the trainee can competently score multiple animals, as recordings can vary significantly between subjects. The trainee is instructed to score across 24 hours to observe both light and dark-phase sleep and wake patterns.

# The trainee will score each 24-hour recording until the expert deems it satisfactory. During the first attempt, the trainee is encouraged to use the expert scoring as a reference. During subsequent attempts, the trainee scores the 24-hour recording again from scratch but is encouraged not to use the expert scoring as a reference. After each attempt, the expert reviews the scorings with the trainee. The expert will assess vigilance state durations, percentage of time spent in each vigilance state, bout numbers and vigilance state transitions during both the light and dark phase. Selected sections of a recording period will be evaluated to provide feedback on transitions between vigilance states. Correlation analysis between the expert and trainee is conducted across parameters before graduating a trainee to expert status.

# Figure Labels

**Figure S1. Diagram of dataset creation.** PSG data acquired for 24 hours for each of 16 rats produced 16 discrete 24-hour PSG recordings. The same 16 rats underwent PSG data acquisition for another 24 hours, producing another set of 16 discrete 24-hour PSG recordings. Rat identity was maintained across these two recording sessions. We grouped both sets of PSG recordings across the rats for proper algorithm training. The grouped set of PSG recordings is shown on the right with 16 vertical rectangles each representing 48 hours of PSG data. We refer to the dataset as 16 48-hour PSG recordings for simplicity.

**Figure S2. Diagram of fundamental components of our algorithm.** (A) The 10-second epoch encoder, and (B) the residual block. The 10-second epoch encoder consists of a sequence of residual blocks and then global average pooling. Each residual block has a number that denotes the number of latent feature maps to use in that block. The residual block consists of a sequence of convolutions, normalization, and nonlinearities. There is a residual summation before the final nonlinearity. Conv1d layers have a number that denotes the kernel size of convolution.

**Figure S3. Comparison between sleep stages in our prediction signal and the SPINDLE reference signal.** Our network predicts sleep stages in 10-second epochs, but the SPINDLE dataset provides a reference in 4-second epochs. Therefore, we devised a reasonable method of comparison shown here. Without loss of generality, we represented two different sleep stages (they may be any two) by vertical lines and horizontal lines, respectively. For each reference 4-second epoch, the epoch was either contained within our prediction 10-second epoch or evenly spanned two of our prediction 10-second epochs. For each reference 4-second epoch entirely contained within a prediction 10-second epoch, we compared these sleep stages directly. For the reference 4-second epoch that evenly spanned two of our prediction 10-second epochs, we evaluated average correctness over the two prediction 10-second epochs. For example, (A) if an S reference 4-second epoch spanned an S-P pair of prediction 10-second epochs, we assigned an accuracy of 0.5. (B) If a P reference 4-seconds epoch spanned a P-P pair of prediction 10-second epochs, we assigned an accuracy of 1. (C) If the prediction 10-second epochs **were** both incorrect, we assigned an accuracy of 0. S – NREM/slow wave sleep, P – REM/paradoxical sleep.

**Figure S4. Performance of our algorithm on parameters of the testing set.** Panels (A-C) show the total bout duration for REM/Paradoxical Sleep, NREM/Slow Wave Sleep, and Wakefulness, respectively, against predicted values. Panels (D-F) display the average bout duration for REM/Paradoxical Sleep, NREM/Slow Wave Sleep, and Wakefulness, respectively, against predicted values. Panels (G-I) present the total number of bouts for REM/Paradoxical Sleep, NREM/Slow Wave Sleep, and Wakefulness, respectively, against predicted values. Linear regression was applied for all combinations of sleep stages and parameters. The shading represents the 95% confidence interval, and the x-axis shows the predicted values while the y-axis shows the measured (or reference) values. The analysis demonstrates the model’s ability to predict bout parameters with reasonable confidence, with tighter fits in some stages and parameters compared to others.

**Figure S5. Performance of the algorithm on the SPINDLE dataset over 22 folds of cross-validation.** (A) Precision, recall, and F1-score over 22 folds of cross-validation are shown by a boxplot. Individual outliers are denoted with open circles. (B) Correlation between macro F1-score and average confidence levels of the algorithm. Overall confidence was calculated for each EEG recording by averaging the confidence levels across all 10-second epochs. (C) Confusion matrix. The diagonal elements represent the percentage of 10-second epochs that were correctly classified by the algorithm (recall), whereas the off-diagonal elements show the percentage of 10-second epochs mislabeled by the algorithm. (D) Duration of each sleep stage for predicted signal and reference signal over 16 folds of cross-validation expressed as a proportion of the total duration of the given EEG recording. Pairs of boxplots are shown for each stage where the left box depicts the predicted distribution, and the right box depicts the reference distribution. P – REM/paradoxical sleep, S – NREM/slow wave sleep, W - wakefulness.

**Figure S6. Stacked representation of algorithm predictions, raw data, and algorithm confidence for one fold.** (A) The reference hypnogram as annotated by a human expert. (B) The predicted hypnogram as predicted by the algorithm. (C) The raw EEG signal that is used as the sole network input. (D) The probability distribution over sleep stages produced by the network. Additionally, the confidence level of the network is denoted by black line. P – REM/paradoxical sleep, S – NREM/slow wave sleep, W - wakefulness.

**Figure S7. Concordance of sleep-wake classification of SnoozyRat dataset by LSTM scoring and two human experts.** Vigilance state durations were evaluated in 1-hr bins during the light phase (Zeitgeber Time (ZT) 0 – 12) and dark phase (ZT 12-24). LSTM and human experts classified more NREM and REM sleep during the light phase and more wake during the dark phase. (A) NREM sleep duration in 1- hr bins. (B) Total NREM sleep duration by phase. (C) REM sleep duration in 1-hr bins. (D) Total REM sleep duration by phase. (E) Wake duration in 1-hr bins. (F) Total Wake duration by phase. RM ANOVA with Bonferroni’s post-hoc test. * P < 0.05, **P < 0.01. Data are mean ± SEM. N=6/group.

**Figure S8. Significant correlations of vigilance state durations across 24-hours:** **LSTM Scoring vs. Human experts and inter-rater agreement among experts**. Correlation analysis was conducted with SnoozyRat dataset to evaluate vigilance state durations between LSTM scoring and human experts, as well as among human experts. (A-C) NREM sleep duration. (D-F) REM sleep durations. (G-I) Wake duration. Linear regression was applied for all analyses and R^2^ greater than 0.94 for all analyses demonstrated the LSTM model’s ability to predict vigilance state durations and high inter-rater agreement between human experts.

**Figure S9. Significant correlations of vigilance state bout numbers across 24-hours:** **LSTM Scoring vs. Human experts and inter-rater agreement among experts**. Correlation analysis was conducted with SnoozyRat dataset to evaluate vigilance state bout numbers between LSTM scoring and human experts, as well as among human experts. (A-C) NREM sleep bouts. (D-F) REM sleep bouts. (G-I) Wake bouts. Linear regression was applied for all analyses and demonstrated the LSTM model’s ability to predict vigilance state bout numbers and the inter-rater agreement between human experts.

**Figure S10. Significant correlations of vigilance state average bout durations across 24-hours:** **LSTM Scoring vs. Human experts and inter-rater agreement among experts**. Correlation analysis was conducted with SnoozyRat dataset to evaluate vigilance state average bout durations between LSTM scoring and human experts, as well as among human experts. (A-C) NREM sleep average bout durations. (D-F) REM sleep average bout durations. (G-I) Wake average bout durations. Linear regression was applied for all analyses and demonstrated the LSTM model’s ability to predict vigilance state bout numbers and the inter-rater agreement between human experts.

**Figure S11: Sleep-wake duration and architecture data do not significantly differ between LSTM and human expert scoring of SleepyRat dataset.** (A) NREM duration, (B) REM duration, (C) Wake duration, (D) NREM bout number, (E) REM bout number, (F) Wake bout number, (G) average NREM bout duration, (H) average REM bout duration, (I) average Wake bout duration. Two-way RM ANOVA with post-hoc Dunnett test. * P < 0.05, ** P < 0.01, *** P < 0.001, **** P < 0.0001. Data are mean ±SEM. N =14 per group.
